# MEIS1::NCOA2 Fusion Sarcoma of the Bartholin Gland: A Case Report and Review of the Literature

**DOI:** 10.3390/diagnostics16132098

**Published:** 2026-07-03

**Authors:** Pauline Dumonceaux, Loréane Sims, Aline Francois, Sabrina Croce, Latifa Fellah, Sophie Cvilic, Charlotte Maillard, Pascale Jadoul

**Affiliations:** 1 Department of Gynecology, Cliniques Universitaires Saint-Luc, 1200 Brussels, Belgium; pauline.dumonceaux@student.uclouvain.be (P.D.); loreane.sims@student.uclouvain.be (L.S.); charlotte.maillard@saintluc.uclouvain.be (C.M.); 2 Department of Anatomical Pathology, Saint-Luc University, 1200 Brussels, Belgium; a.francois@saintluc.uclouvain.be; 3 Department of Biopathology, Institut Bergonié, 33000 Bordeaux, France; s.croce@bordeaux.unicancer.fr; 4 Department of Radiology, Saint-Luc University, 1200 Brussels, Belgium; latifa.fellah@saintluc.uclouvain.be; 5 Department of Radiation, Oncology Saint-Luc University, 1200 Brussels, Belgium; sophie.cvilic@saintluc.uclouvain.be

**Keywords:** MEIS1::NCOA2 fusion, Bartholin gland tumor, vulvar sarcoma, CTNNB1

## Abstract

**Background:** MEIS1::NCOA1/2 fusion sarcomas are a recently described molecular entity arising predominantly in the genitourinary and gynecologic tracts. Their clinical presentation is often misleading, and no standardized treatment guidelines currently exist. **Methods:** A literature review was conducted using PubMed to identify all reported cases of molecularly confirmed MEIS1::NCOA1/2 fusion sarcomas. Clinicopathological, molecular, treatment, and outcome data were extracted for comparative analysis. **Case:** We report the case of a 38-year-old nulliparous woman who presented with a right vulvar induration clinically consistent with a Bartholin gland cyst. Surgical excision revealed a spindle cell mesenchymal tumor harboring a MEIS1::NCOA2 fusion transcript and a CTNNB1 exon 3 mutation, with probable incomplete resection margins. A local recurrence was documented by MRI and PET–CT at eight months. Surgical re-excision revealed diffuse involvement and complete excision was considered uncertain. Adjuvant external beam radiotherapy followed by an MR-Linac boost was administered. **Discussion:** This case highlights the diagnostic challenge of Bartholin gland masses. We provide a review of the literature on MEIS1::NCOA1/2 fusion sarcomas and examine the potential aggressiveness of tumors that additionally harbor a CTNNB1 mutation. Given the nonspecific immunophenotype of this entity, this case underscores the indispensable role of RNA-based molecular sequencing in the diagnosis of low-grade spindle cell tumors when immunohistochemistry proves inconclusive. We further discuss the surgical challenges inherent to this anatomical region and explore intention-to-treat radiotherapy as a potential therapeutic option. **Conclusions:** We report a rare case of a MEIS1::NCOA2 fusion-positive sarcoma arising in the Bartholin gland region, and, to our knowledge, the first case in which radiotherapy with curative intent has been explored for this entity. This observation expands the limited literature on this emerging clinicopathological entity.

## 1. Introduction

Bartholin gland lesions are a common gynecologic complaint, accounting for approximately 2% of all gynecologic visits annually [1]. The vast majority are benign—cysts, abscesses, and are managed based on symptomatology and patient age [2]. Malignant tumors of the Bartholin gland are exceptional, comprising only 0.1 to 5% of all vulvar carcinomas and an estimated 0.001% of all female malignancies [3]. Among these, adenocarcinomas and squamous cell carcinomas predominate, while sarcomas represent a minor subset of Bartholin gland malignancies, with only a handful of cases documented in the literature [4].

Bartholin gland tumors are frequently mistaken for benign diseases, creating a significant risk of diagnostic delay [3,4]. Several series and case reports have documented this pitfall: leiomyosarcomas [5], epithelioid sarcomas [6], and other mesenchymal tumors in the Bartholin gland region have been initially misidentified as cysts or abscesses. Current clinical practice recommends biopsy or excision of Bartholin gland masses primarily in the presence of atypical features or in patients over 40 years of age [3]; however, these criteria do not capture all cases of underlying malignancy, particularly in young patients.

MEIS1::NCOA1/2 fusion sarcomas constitute a newly described molecular entity, first reported in 2018 in two cases of primitive renal spindle cell sarcoma [7]. Subsequent reports have expanded the anatomical spectrum to include the uterine corpus, vagina, scrotum, para-rectal region, pelvis, and lung [8,9,10,11,12]. These tumors are characterized by a nonspecific immunophenotype, frequent local recurrences, and generally low-grade clinical behavior, although at least six cases with distant metastatic progression have now been documented [11,13,14,15,16]. Accurate diagnosis requires RNA-based molecular testing, as histological and immunohistochemical characteristics can overlap with other spindle cell neoplasms, including low-grade endometrial stromal sarcomas [8,9].

We report the first case of a MEIS1::NCOA2 fusion sarcoma arising in the Bartholin gland region, in a young nulliparous woman initially evaluated for a benign-appearing vulvovaginal symptomatic mass. This case illustrates the diagnostic trap inherent to Bartholin gland masses, the indispensable role of molecular analysis in the classification of vulvar mesenchymal tumors, and the complex oncological management of this emerging entity at an anatomically challenging site.

## 2. Methods

A review of the literature was performed using the PubMed database. To identify all previously reported cases of MEIS1::NCOA1/2 fusion sarcomas, the following search queries were used: “MEIS1 AND NCOA2 AND sarcoma”, “MEIS1 AND NCOA1 AND sarcoma”, and “MEIS1 AND NCOA1/2 AND fusion”. Cases were included if they reported a molecularly confirmed MEIS1::NCOA1 or MEIS1::NCOA2 fusion. For each case, available data on tumor location, fusion partner, CTNNB1 status, associated genomic alterations, treatment, recurrence or metastasis, and clinical outcome were extracted to enable comparative analysis. To contextualize the rarity of sarcomas at this anatomical site, an additional search was performed using “Bartholin gland AND sarcoma”. The reference lists of all retrieved articles were manually screened to identify additional relevant cases. Articles published in English and French up to March 2026 were considered.

## 3. Case Presentation

### 3.1. Initial Presentation and Surgical Management

A 38-year-old nulliparous woman presented to the gynecology outpatient clinic for advice on a known fibroid and suspicion of endometriosis. She mentioned an 8-year history of regular vulvovaginal pain radiating toward the anus, associated with introital dyspareunia for which she received the diagnosis of pudendal nerve pain and had been undergoing pelvic floor physiotherapy. Her medical history was notable for congenital factor XII deficiency; she had no prior relevant gynecological history and was using a vaginal ring (NuvaRing, Organon, The Netherlands) continuously for contraception, resulting in amenorrhea.

Physical examination revealed a right vulvar induration measuring approximately 1.5 cm, located in the anatomical region of the Bartholin gland, which was tender on palpation. No inguinal lymphadenopathy was detected. The clinical presentation was consistent with a Bartholin gland cyst, and surgical excision was proposed given the significant functional impairment. Concurrent with this consultation, a pelvic ultrasound identified an anterior FIGO type 2 fibroid of 22 mm; hysteroscopic myomectomy was therefore performed in the same operative session.

Intraoperatively, palpation revealed a deep mass of approximately 3.5 cm within the Bartholin gland region, extending posteriorly toward the ischiopubic ramus—an extent not anticipated from the preoperative clinical assessment. The lesion appeared solid, pinkish, with millimetric mucosal excrescences and small mural indurations noted on its surface. Excision was macroscopically complete. The specimen was sent for routine histopathological analysis.

### 3.2. Pathological Findings

Gross examination revealed a fragmented solid mass measuring 3.5 × 1 × 1 cm. Histopathological analysis demonstrated a heterogeneous mesenchymal nodular proliferation composed of mildly atypical spindle cells arranged in interlacing fascicles. The tumor cells exhibited predominantly oval, relatively uniform nuclei, with occasional hyperchromasia. The cytoplasm was generally pale and ill-defined, with focal areas showing a more eosinophilic appearance. In certain regions, the cells formed whorled architectures with alternating cellularity and displayed a focally epithelioid morphology (Figure 1a). Mitotic activity was low, with fewer than 1 mitosis per 10 high-power fields (HPF, ×400). No tumor necrosis was identified. Foci of stromal edema and areas of hemorrhagic extravasation were present (Figure 1b). The vascular network consisted predominantly of delicate, thin-walled vessels, often elongated and occasionally exhibiting a pseudo-hemangiopericytomatous pattern. Less frequently, vessels with thicker, arteriole-like walls were observed. The lesion was noted to be in intimate contact with the mucinous glandular acini of the Bartholin gland, into which it insinuated and separated the native structures without overt destructive invasion (Figure 1c). Given the fragmented nature of the specimen, the tumor borders were difficult to appraise, and the completeness of surgical excision could not be reliably assessed, although it was considered incomplete.

Immunohistochemical studies showed a nonspecific and heterogeneous profile. CD10 expression was observed in areas with whorled architecture, whereas desmin and smooth muscle actin displayed a complementary (“mirror”) staining pattern (Figure 1d,f). The tumor cells were negative for cytokeratin AE1/AE3, H-caldesmon, HMB45, STAT6, CD34, S100 protein, EMA, and CD68. Estrogen receptor (ER) demonstrated moderate to strong diffuse nuclear positivity, while progesterone receptor (PR) expression was heterogeneous. Given the partial expression of smooth muscle markers, an FH-deficient leiomyoma was considered in the differential diagnosis and excluded based on the negativity of S-(2-succino)-cysteine (2SC) and retained expression of fumarate hydratase (FH).

There was no immunoreactivity for myogenin, excluding the possibility of rhabdomyosarcoma. An inflammatory myofibroblastic tumor (IMT) was also excluded by ALK immunohistochemistry. Nuclear accumulation of β-catenin was identified in the whorled regions, suggesting an exon 3 mutation of CTNNB1, which was later confirmed by next-generation DNA sequencing (Figure 1g).

MDM2 showed weak and focal nuclear staining, and MDM2 fluorescence in situ hybridization (FISH) was later performed.

Retinoblastoma protein (pRB) demonstrated weak and heterogeneous staining (considered retained). p16 expression showed a non–block-type heterogeneous pattern with increased intensity in the whorled areas. p53 exhibited a wild-type staining pattern with heterogeneous nuclear intensity and distribution. Pan-TRK showed weak heterogeneous cytoplasmic staining, considered inconclusive. Cyclin D1 was negative (cut off 70% of nuclei stained), and BCOR was negative. INI1 (BAF47) and SMARCA4 expression were retained.

Molecular analysis by whole RNA sequencing identified a MEIS1::NCOA2 fusion transcript: exon 7 of MEIS1 (NM_002398.3) fused to exon 13 of NCOA2 (NM_006540.4). The sample showed clustering with a group of MEIS1-rearranged neoplasms/sarcomas.

SNP-array–based genomic profiling showed a flat genomic profile, consistent with low genomic complexity. In the context of a simple-genomics sarcoma, this profile would be classified as copy-number variation–low (CNV-low).

A likely pathogenic CTNNB1 mutation was identified in exon 3 (p.Ser33Cys, c.98C > G; VAF 8.1%) by DNA-based next-generation sequencing. No MDM2 amplification was identified by either FISH analysis or SNP-array-based genomic profiling.

The overall histopathological picture was therefore consistent with a sarcoma harboring a MEIS1::NCOA2 fusion. The resection was considered incomplete (enucleation with suspected positive margins).

Molecular analyses were performed on formalin-fixed, paraffin-embedded (FFPE) tissue after macrodissection of tumor-rich areas. RNA-based next-generation sequencing (NGS) was performed using the FusionPlex^®^ Sarcoma V2 panel (ArcherDX 7.4.3) on a MiSeq platform (Illumina, Brussels, Belgium). Sequencing data were analyzed using Archer Analysis software (ArcherDX 7.4.3) for the detection of gene fusions. For DNA-based NGS, genomic DNA was extracted and analyzed using the custom capture panel TE-98036185_hg38 (Twist Bioscience, South San Francisco, CA, USA) on a NextSeq platform (Illumina, Brussels, Belgium). Variant calling and interpretation were performed using the Sophia DDM^®^ platform (Sophia Genetics, Saint-Sulpice, Switzerland, 7.18.0). MDM2 amplification status was assessed by fluorescence in situ hybridization (FISH) using the Vysis MDM2/CEP12 Probe Kit (Abbott Molecular, Brussels, Belgium) and SNP-array CGH (Bordeaux, France).

In addition, genome-wide copy number analysis was performed using the OncoScan^®^ CNV FFPE Assay (Affymetrix/Thermo Fisher Scientific, Waltham, MA, USA), with array scanning carried out on a GeneChip^®^ Scanner 7G. For total RNA sequencing, RNA was extracted using the Maxwell^®^ RSC RNA FFPE Kit (Promega, Madison, WI, USA), including DNase treatment, and RNA quality was assessed using an Agilent Bioanalyzer (Agilent Technologies, Santa Clara, CA, USA) with the RNA Nano assay. Library preparation was performed using the TruSeq RNA Exome kit (Illumina, Bordeaux, France), and sequencing was carried out on a NextSeq 500 platform (Illumina, Bordeaux, France).

### 3.3. Staging and Multidisciplinary Management

Following the unexpected diagnosis, a staging workup was performed including chest X-ray, thoracoabdominal CT scan, and pelvic MRI. The pelvic MRI showed no evidence of local residual tumor at the surgical site. Both CT-scan and pelvic MRI identified indeterminate osteocondensing iliac lesions (largest 25 mm on the left), assessed as likely non-evolutive. No distant metastases were identified.

The multidisciplinary oncology tumor board recommended surgical re-excision to achieve clean margins. The patient declined further surgery at that time and opted for close clinical and radiological surveillance with pelvic MRI and chest X-ray every 6 months.

### 3.4. Local Recurrence and Second Surgery

At 8-month follow-up (delayed from the planned 6-month interval for logistical reasons), pelvic MRI demonstrated a 22 × 13 mm diffusion-restricted lesion located superior to the Bartholin gland region surgical scar, strongly suspicious for local recurrence, without associated lymphadenopathy (Figure 2).

Chest X-ray was normal. A subsequent PET–CT confirmed a mildly hypermetabolic focus at this site, consistent with sarcoma recurrence.

The case was reviewed by the multidisciplinary pelvic oncology tumor board, which recommended wide local re-excision. Surgery was performed approximately 10 months after the initial operation, with multiple margin resections (antero-superior, superficial superior, deep).

Histological analysis confirmed diffuse residual MEIS1::NCOA2 fusion sarcoma, with tumor foci measuring up to 10 mm and attaining the inked surgical margin (Figure 3). No lymphovascular tumor emboli were identified. Resection was estimated as incomplete. A further surgical attempt was collectively judged excessively morbid given the proximity of critical perineal structures.

### 3.5. Adjuvant Treatment and Current Status

The sarcoma multidisciplinary board recommended radiotherapy, based on extrapolation from established soft tissue sarcoma management principles, particularly in the context of two R1 resections and the absence of entity-specific treatment guidelines for this rare tumor type. The radiation oncology team confirmed that the planned irradiation field would remain localized to the vulvar region, without irradiation of the ovaries or uterus. Fertility preservation was discussed with the patient; hormonal workup demonstrated a favorable ovarian reserve (AMH 10.5 ng/mL). After counseling by the fertility team, the patient ultimately declined oocyte cryopreservation.

At the time of writing, the patient has completed external beam radiotherapy, consisting of 50 Gy delivered in 25 fractions of 2 Gy to the tumor bed with a 3 cm margin, modified to respect anatomical barriers using an Ethos accelerator (Varian Medical Systems, Siemens Healthineers Company, Palo Alto, CA, USA). A boost dose of 20 Gy in 5 fractions (5 × 4 Gy) was prescribed to the tumor bed using an MR-Linac Monaco v.6.2.2.0 (Elekta Ltd. in Stockholm, Sweden). She is with a total follow-up of 15 months from initial diagnosis. Regarding post-treatment surveillance, given the imaging modalities that detected recurrence, follow-up was planned with alternating PET–CT and pelvic MRI every 6 months, complemented by clinical examinations every 3 months during the first 2 years.

## 4. Discussion

### 4.1. The Bartholin Gland as a Diagnostic Pitfall for Rare Malignancies

Bartholin gland lesions are predominantly benign, and their management is largely guided by clinical appearance and symptomatology. This diagnostic method, while efficient for the vast majority of cases, creates a risk of missed or delayed diagnoses of rare malignancies. The present case illustrates this risk: a 38-year-old woman with a small (1.5 cm), tender, unilateral vulvar induration, clinically resembling a simple cyst, was ultimately found to be a locally aggressive mesenchymal tumor. This diagnostic trap is well documented in the literature. As early as 1994, Konefka et al. described a case of epithelioid sarcoma of the Bartholin gland region initially misdiagnosed as vulvar carcinoma, ultimately resulting in pulmonary metastases and death within 8 months despite aggressive treatment [6]. González-Bugatto et al. reported a vulvar leiomyosarcoma in the Bartholin gland area presenting as a benign cystic lesion in a 52-year-old woman [5]. Saquib et al. reported a leiomyosarcoma mimicking a chronic Bartholin cyst in a 63-year-old postmenopausal woman [17], while Reinicke et al. described an identical diagnostic pitfall in a 14-year-old adolescent [18]—showing that this trap applies across the entire age spectrum.

This raises the question of whether additional triggers—such as unusual depth on palpation, extension beyond expected gland boundaries, or a clinical evolution disproportionate to the presumed benign diagnosis—should prompt a biopsy or imaging.

### 4.2. The Potential Role of Preoperative Imaging

A reflection point in our case is the absence of preoperative imaging beyond a routine pelvic ultrasound. The intraoperative discovery of a 3.5 cm mass extending to the ischiopubic ramus, not appreciated on clinical examination, could potentially have been identified by preoperative transperineal ultrasound or MRI, possibly allowing a planned wide local excision.

T2-weighted pelvic MRI is the imaging modality of choice for confirmed or suspected Bartholin gland carcinomas, providing information on proximity to the local surrounding structures, and presence of lymphadenopathy [19]. We acknowledge that MRI is not routinely indicated for the evaluation of a presumed Bartholin gland cyst. However, our case suggests that additional imaging may be considered in selected patients with atypical clinical features, such as unusual depth, firmness, fixation to adjacent structures, recurrent lesions, or extension inconsistent with a simple cyst. In such situations, preoperative MRI may better delineate the lesion and facilitate appropriate surgical planning.

### 4.3. MEIS1::NCOA2 Fusion Sarcomas: An Emerging Molecular Entity

MEIS1::NCOA2 fusion sarcomas were first described in 2018 by Argani et al. in two cases of primitive spindle cell sarcoma of the kidney [7]. The defining molecular event is a chromosomal rearrangement involving chromosome 2p14 (MEIS1) and chromosome 8q13.3 (NCOA2), producing an in-frame fusion transcript and resulting in a chimeric protein that combines the DNA-binding homeodomain of MEIS1—a TALE-family transcription factor—with the transcriptional activation domains of NCOA2, nuclear hormone receptor transcriptional coactivator. This fusion transcript is detected at the RNA level by next-generation sequencing [7,8].

Kao et al. expanded the clinicopathological spectrum in 2021, reporting six additional cases involving the kidney, uterine corpus, vagina, scrotum, and para-rectal region, and suggesting gynecologic and genitourinary tract as the preferential anatomical territory [8]. Since then, cases have been reported in the uterus [10,11,13], fallopian tube [12], and pelvic cavity [14,20]. In parallel, a closely related variant fusion involving the nuclear receptor coactivator NCOA1 was first identified in a renal case in the Kao study [8], and subsequently described in a uterine sarcoma [9] and a pelvic sarcoma [20]. In 2025, Argani et al. reported 7 additional primary primitive renal sarcomas with a MEIS1::NCOA1 gene fusion [16]. Their clinical, morphological and immunohistochemical features overlapped with the previously described MEIS1::NCOA2 renal sarcomas. On this basis, the authors propose grouping these neoplasms together as MEIS1::NCOA1/2 primitive sarcomas.

The present case extends this spectrum to the Bartholin gland region, representing—to our knowledge—the first documented MEIS1::NCOA2 sarcoma at this location. Including this one, approximately 25 cases of primitive MEIS1::NCOA1/2 sarcomas of the genitourinary tract have been reported to date [7,8,9,10,11,12,13,14,15,16,20]. The clinical profile reveals female predominance across a wide age range, frequently presenting local recurrences. We have identified distant metastatic progression documented in at least 6 cases [11,13,14,15,16]. A comparative summary of all published genitourinary-tract MEIS1::NCOA1/2 fusion sarcomas reported to date, together with the present case, is provided in Table A1.

The immunophenotype is nonspecific, with variable expression of cyclin D1, CD56, CD10, ER, PR [8,16].

It is important to note that the MEIS1::NCOA1/2 fusion is not exclusive to the genitourinary spectrum. The same fusion has been identified in intraosseous spindle cell rhabdomyosarcomas—with a more aggressive clinical behavior [21] and in a primary pulmonary spindle cell sarcoma behaving in a low-grade indolent fashion [22]. Three distinct anatomical groups have therefore been described to this date, with distinct immunophenotypes and clinical courses, demonstrating that the fusion alone is insufficient to predict behavior.

### 4.4. CTNNB1 Exon 3 Alterations: An Emerging Marker of Aggressive Behavior

The coexistence of a MEIS1::NCOA2 fusion with a CTNNB1 exon 3 alteration in our case warrants specific attention, given a striking pattern that emerges when examining published MEIS1::NCOA1/2 fusion sarcomas of the genitourinary tract with known molecular profiles. Of the five published genitourinary-tract cases carrying a documented CTNNB1 exon 3 alteration, the four with available clinical follow-up displayed aggressive behavior [14,20].

Specifically: Xing et al. reported the first gynecological case with pulmonary metastasis in a uterine sarcoma carrying a CTNNB1 D32H mutation alongside HMGA2/CDK4/MDM2 region amplification [13]; Niu et al. described the first fatal case, a high-grade uterine sarcoma harboring a CTNNB1 H36-T41 deletion alongside amplifications of MDM2, CDK4, MDM4, and FRS2, leading to lung metastasis and patient death at 23 months [11]; Kao et al. reported one uterine case with CTNNB1 p.S33C mutation and MDM2 amplification, which recurred locally at 5 months [8]; and Gao et al. described a pelvic sarcoma with a CTNNB1 p.D32Y mutation alongside 10q23-26 amplifications, which metastasized to the liver and gastroduodenal region with death at 7 months [14]. Notably, Gao et al. observed that all four cases with CTNNB1 alterations coexisted with distinct oncogenic amplifications (12q or 10q), suggesting that CTNNB1 may function with other gene alterations to drive aggressiveness [14].

In our case, the CTNNB1 p.Ser33Cys mutation is present, but MDM2 FISH did not show a gene amplification. The presence of this CTNNB1 alteration warrant vigilant surveillance, but more data are needed to draw conclusions on the real significance of CTNNB1 alterations. Systematic molecular profiling, including MDM2 status and broader copy number analysis should be performed.

### 4.5. Diagnostic and Clinical Parallel with Low-Grade Endometrial Stromal Sarcoma

Our literature review has revealed that the differential diagnosis in the gynecologic setting with low-grade endometrial stromal sarcoma (LG-ESS) can be difficult. Both entities share spindle cell architecture in short fascicles and whorling patterns, low mitotic activity, absence of necrosis, and a similar immunoprofile with expression of CD10, ER, and PR [9,10]. This misclassification is further illustrated by the case of Mejbel et al. in which a MEIS1::NCOA1 uterine sarcoma was initially reported as LG-ESS [9].

From a clinical behavior standpoint, the parallel is also striking: LG-ESS carries a local recurrence rate of up to 54%, even in early stages, a pattern similar to what we observed in MEIS1::NCOA2 sarcomas [23]. This raises the question of whether the therapeutic options should mirror those of LG-ESS. For both entities, complete surgical resection is the logical first step to the treatment when achievable. In LG-ESS, hormonal therapy (aromatase inhibitors or progestins) is an established treatment for recurrent and metastatic disease (recommendation C, level V). Adjuvant radiotherapy may be considered when the risk of local recurrence is deemed significant, though the level of evidence supporting this remains low (recommendation C, level IV) [23]. For MEIS1::NCOA1/2 sarcomas, no therapeutic guidelines exist, and management is currently extrapolated from general soft tissue sarcoma principles.

### 4.6. Surgical Management: The Challenge of Adequate Margins

In the Bartholin gland region, adequate oncological margins are technically challenging. In our case, after the second local re-excision was also suspected to have positive margins, a third surgical attempt was judged excessively morbid, mainly due to the proximity of the ischiopubic ramus. This sequence illustrates a fundamental challenge in anatomically constrained locations.

We question whether the local recurrence observed in our case was primarily due to the incomplete excision or if it was an intrinsic property of the MEIS1::NCOA1/2 sarcoma. We analyzed the published cases presenting local recurrences and margin status was not available in the majority [8,9,14,20]. Quiroga-Garza explicitly stated negative margins after resection of a primary renal sarcoma with MEIS1–NCOA2 fusion and found a local recurrence 4 months after nephrectomy, and pulmonary metastases at 7 months post-op [15], suggesting that recurrence may reflect an intrinsic biological property of this entity, beyond surgical margin status.

### 4.7. Adjuvant Radiotherapy and Emerging Therapeutic Perspectives

Given suspected persistent R1 margins and the anatomical difficulty of further surgery, the multidisciplinary sarcoma board recommended curative-intent adjuvant external beam radiotherapy.

This decision was supported by soft tissue sarcoma and visceral sarcoma guidelines, who recommend adjuvant radiotherapy for patients with positive resection margins [24]. To our knowledge, only one prior case of MEIS1::NCOA1/2 fusion sarcoma treated with radiotherapy has been reported: in the Argani 2025 series, a renal MEIS1::NCOA1 sarcoma with multifocal peritoneal recurrence, in which radiotherapy combined with doxorubicin failed to prevent further progression. The present case represents the first reported use of adjuvant radiotherapy administered in a curative intent in a localized MEIS::NCOA2 sarcoma, following R1 resection.

No systemic chemotherapy was administered, reflecting both the low-grade histological features of the tumor and the limited evidence supporting chemotherapy efficacy in this molecular subtype. Across the literature, responses to chemotherapy have been largely disappointing: in the Argani 2025 series, two patients with advanced or recurrent renal sarcomas (vincristine/doxorubicin/cyclophosphamide; doxorubicin alone) showed no radiological response [16]; Mejbel et al. reported an explicit lack of response to docetaxel [9]; the patient reported by Niu et al. died of disease at 23 months despite chemotherapy [11]; and the patient of Quiroga-Garza et al. progressed (local recurrence at 4 months, lung metastases at 7 months) on a doxorubicin/tazemetostat trial [15]. A notable exception is Xing et al. [13], in whom mesna/doxorubicin/ifosfamide achieved no evidence of disease at 36 months following nodal progression.

Another therapeutic consideration concerns hormonal therapy. The consistent ER and PR expression observed across MEIS1::NCOA1/2 raises the question of whether anti-estrogenic or progestational therapies—well established in of LG-ESS management—could represent a rational therapeutic option in MEIS1::NCOA2 sarcomas as well. To date, the only reported case in which hormonal therapy was administered is that of Mejbel et al., where letrozole was included as part of the treatment regimen for multiple locoregional recurrences; despite this, the patient continued to develop further recurrences and ultimately required systemic chemotherapy [9]. This single experience does not demonstrate a reliable therapeutic effect of hormonal therapy in this entity, although definitive conclusions cannot be drawn from one case. In our case, diffuse intense ER expression and heterogeneous PR positivity theoretically provide a biological rationale for considering this approach should disease progress beyond local control, particularly given the absence of other established systemic therapies.

### 4.8. Fertility Considerations

Given the patient’s nulliparity and young age, the reproductive implications of pelvic radiotherapy were carefully evaluated. The irradiation field was confirmed to exclude the ovaries and uterus, preserving potential future fertility. Oocyte cryopreservation was offered, and ovarian reserve was confirmed to be adequate. The patient ultimately declined after informed counseling. This dimension of care was also recently highlighted by Ramineni et al. in the context of MEIS1::NCOA1 sarcoma [20]. It highlights the importance of fertility counseling in the management of reproductive-age patients with gynecologic sarcomas.

## 5. Conclusions

We report the first case of a MEIS1::NCOA2 fusion sarcoma arising in the Bartholin gland region. The diagnosis was established by RNA-based next-generation sequencing integrating morphological and immunohistochemical features—a pattern now consistently reported across anatomical sites for this entity. The presence of a CTNNB1 alteration warrants vigilant surveillance, although its clinical significance remains to be clarified through further molecular characterization and additional cases. Adjuvant radiotherapy was administered following two R1 resections; to our knowledge, this represents the first reported use of curative-intent radiotherapy in a MEIS1::NCOA1/2 fusion sarcoma. Given the rarity of this entity and the lack of entity-specific management recommendations, this treatment approach was extrapolated from established soft tissue sarcoma principles. As the case count of this rare entity slowly grows, further clinical and molecular observations will contribute to risk stratification and the development of evidence-based management strategies for this emerging entity.

## Figures and Tables

**Figure 1:**
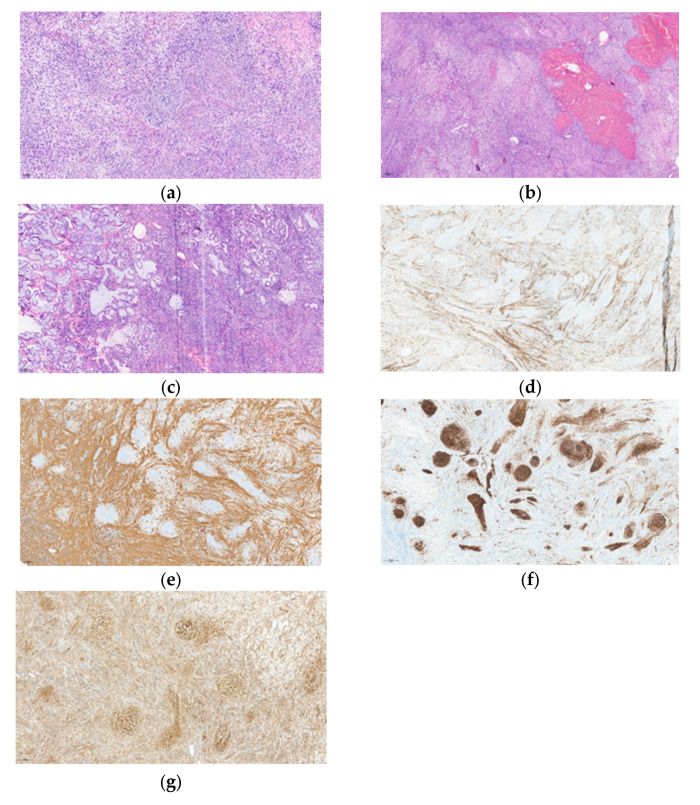
(**a**) Hematoxylin and eosin (H&E), ×13.6: Interlacing fascicles of spindle cells with a whorled architectural pattern; (**b**) Hematoxylin and eosin (H&E), ×6.2: Alternating areas of hypercellularity and hypocellularity with associated hemorrhage; (**c**) Hematoxylin and eosin (H&E), ×12.4: Tumor cells dissecting between Bartholin gland mucinous acini without evidence of destructive invasion; (**d**) Desmin immunohistochemistry, ×6.3: Focal expression within fascicular areas, with absence of staining in whorled regions; (**e**) α–smooth muscle actin (α-SMA) immunohistochemistry, ×7.7: Positive staining in fascicular areas and negative staining in whorled regions; (**f**) CD10 immunohistochemistry, ×8.4: Intense staining in the whorled areas, mirroring the smooth muscle markers; (**g**) β-catenin immunohistochemistry, ×17.2: Predominantly nuclear expression in whorled areas, with cytoplasmic and membranous staining in fascicular regions.

**Figure 2:**
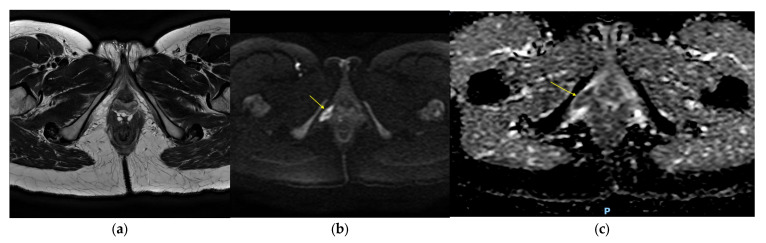
(**a**) Axial T2-weighted MRI image, no lesion identified (anatomical landmark). (**b**) Axial diffusion-weighted MRI image shows high signal intensity at the resection site (between the vaginal and the ischiopubic ramus). (**c**) The apparent diffusion coefficient (ADC) shows low signal intensity and diffusion restriction.

**Figure 3:**
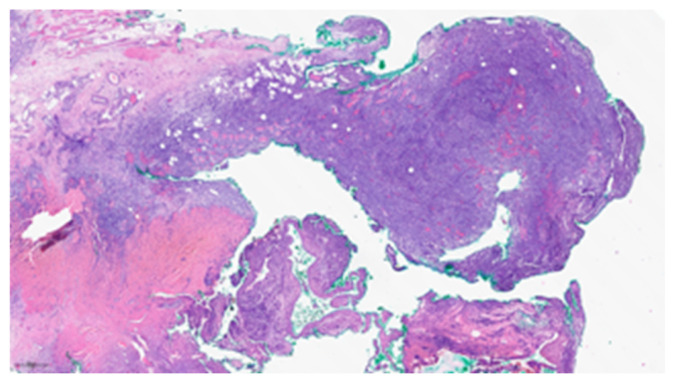
Hematoxylin and eosin (H&E), ×3.8: Recurrent lesion showing infiltrative growth with poorly defined borders, dissecting skeletal muscle fibers and adipose tissue.

## Data Availability

The original contributions presented in this study are included in the article. Further inquiries can be directed to the corresponding author.

## Abbreviations

The following abbreviations are used in this manuscript:

H&E	Hematoxylin and Eosin
FISH	Fluorescence In Situ Hybridization
MRI	Magnetic Resonance Imaging
PET–CT	Positron Emission Tomography—Computed Tomography
CT	Computed Tomography
X-ray	X-ray Imaging
ADC	Apparent Diffusion Coefficient
IMT	Inflammatory Myofibroblastic Tumor
LG-ESS	Low-Grade Endometrial Stromal Sarcoma
FIGO	International Federation of Gynecology and Obstetrics
R1	Microscopically Positive Resection Margin
Gy	Gray
AMH	Anti-Müllerian Hormone

## Appendix A

**Table A1:** Reported cases of primitive MEIS1::NCOA1/2 fusion sarcomas: clinicopathological, molecular, treatment and outcome data.

Study (Year)	Age/Sex	Location/Size	Fusion (Exons)	CTNNB1 Status	Other Genomic Alterations	Primary Treatment	Recurrence/Metastasis	Treatment at Recurrence/Metastasis	Outcome/Survival (Follow-Up)
Renal									
Argani et al. 2018 [7] (Case 1)	72/F	Kidney (left); 4 cm, cystic	MEIS1 ex7::NCOA2 ex13/14	Not reported	None reported	Partial nephrectomy	None	—	No evidence of disease (9 mo)
Argani et al. 2018) [7] (Case 2)	21/M	Kidney (left); 15 cm, solid	MEIS1::NCOA2 (FISH)	Not reported	None reported	Radical nephrectomy	None documented	—	Not available (no follow-up)
Kao et al. 2021 [8] (Case 5)	20/F	Kidney; 21 cm	MEIS1 ex7::NCOA1 ex13	Not tested (fusion panel)	MDM2 negative (FISH)	Not detailed	No follow-up available	—	Not reported (no follow-up)
Kao et al. 2021 [8] (Case 6)	50/F	Kidney (+2 soft-tissue implants); 19 cm	MEIS1 ex7::NCOA2 ex14	Not tested (fusion panel)	MDM2 negative	Total nephrectomy + 10th-rib resection	Local recurrence retroperitoneum at 1.5 y; retrocaval + chest wall/diaphragm at 6 y	Not detailed	Alive, no distant spread/death (≥6 y)
Quiroga-Garza et al. 2021 [15]	51/F	Kidney (left); 19 cm	MEIS1 ex6::NCOA2 ex12	Not reported	MDM2 negative (FISH)	Radical nephrectomy + adrenalectomy (negative margins)	Local recurrence at 4 mo; lung metastases at 7 mo	Doxorubicin + tazemetostat (phase IB trial)	Alive, on treatment (~7 mo)
Argani et al. 2025 [16] (Case 1)	51/M	Kidney (right); 5.3 cm	MEIS1 ex7::NCOA1 ex15	Not reported	None reported	Complete resection	Not reported	—	Not reported (no follow-up)
Argani et al. 2025 [16] (Case 2)	35/F	Kidney (left); 4.9 cm	MEIS1 ex7::NCOA1 ex15	Not reported	None reported	Complete resection (organ-confined)	Not reported	—	Not reported (no follow-up)
Argani et al. 2025 [16] (Case 3)	5/M	Kidney (right); 6.5 cm	MEIS1 ex7::NCOA1 ex15	Not reported	None reported	Nephrectomy	No recurrence at 5 y; retroperitoneal + lung metastases at 9 y	Incomplete retroperitoneal resection + VAC chemotherapy + lung resection	Alive with disease (>9 y)
Argani et al. 2025 [16] (Case 4)	49/M	Kidney (left); 28 cm	MEIS1 ex7::NCOA1 ex15	Mutated D32Y (β-catenin IHC negative)	*ATM* W1221fs * 2	Resection	Not reported	—	Not reported (no follow-up)
Argani et al. 2025 [16] (Case 5)	22/F	Kidney (right); 25.7 cm	MEIS1 ex6::NCOA1 ex12	Not reported	INI1/BRG retained	Resection	Not reported	—	Not reported (no follow-up)
Argani et al. 2025 [16] (Case 6)	32/M	Kidney (left); 26 cm	MEIS1 ex6::NCOA1 ex13	Not reported	None reported	Resection	Multifocal abdominal recurrence + hemoperitoneum at 3 mo; peritoneal recurrences; inguinal hernia metastasis	Radiotherapy * + doxorubicin + serial paracentesis	Dead of disease (9 mo)
Argani et al. 2025 [16] (Case 7)	8/M	Kidney (side not specified); 25 cm	MEIS1 ex7::NCOA1 ex15	Not reported	None reported	Incomplete resection; no adjuvant therapy	Not detailed	—	Died (1 y; presumed tumor, unconfirmed)
Uterine corpus									
Kao et al. 2021 [8] (Case 1)	58/F	Uterine corpus; 20 cm	MEIS1 ex7::NCOA2 ex13	Mutated S33C (β-catenin nuclear+)	*MDM2* amplification; *ATRX* C280fs * 3; *MPL* Y252H; TMB 3 mut/Mb	Total hysterectomy + BSO (intra-op rupture)	Local recurrence on mesentery at 5 mo; no distant spread	Not detailed	Alive (5 mo)
Kommoss et al. 2022 [10]	26/F	Uterine corpus; 9 cm	MEIS1 ex6::NCOA2 ex12	Not tested	No amplification/deletion.	Simple hysterectomy (adnexa preserved); no adjuvant	None	—	No evidence of disease (9 y)
Mejbel et al. 2023 [9]	62/F	Uterus, right pelvic sidewall, right round ligament; 3 cm	MEIS1 ex7::NCOA1 ex13	Not tested (CTNNB1 not in 94-gene fusion panel)	None reported	Total hysterectomy +BSO + adjuvant letrozole	Recurrence at 24 mo (peritoneum, omentum, mesentery, pelvic sidewall); advanced multivisceral recurrence at 36 mo	Surgical resection(s) + docetaxel	Alive but repeatedly recurrent (≥36 mo)
Niu et al. 2023 [11]	41/F	Uterine corpus; 13 cm	MEIS1::NCOA2 (exons not specified)	Mutated H36–T41 deletion (*CTNNB1* exon 3)	*MDM2*, *CDK4*, *MDM4*, *FRS2* amplification (12q13-15)	Total hysterectomy + BSO + omentectomy + peritoneal debulking	Local recurrence on vaginal cuff & pelvis; lung metastasis	Multiple chemotherapy cycles	Dead of disease (23 mo)
Xing et al. 2024 [13]	47/F	Uterine corpus; 7.8 cm	MEIS1 ex7::NCOA2 ex14	Mutated D32H (β-catenin nuclear +)	*HMGA2* + *CDK4* amplification; *MDM2* marginal amplification	TAH + bilateral oophorectomy; no adjuvant	Lung metastasis at 27 mo (nodule from 12 mo); mediastinal/hilar nodes at 30 mo	Lung metastasectomy + 6 cycles mesna/doxorubicin/ifosfamide	No evidence of disease (36 mo; after chemotherapy)
Other anatomical sites									
Kao et al. 2021 [8] (Case 2)	35/F	Vagina; 11 cm	MEIS1 ex6::NCOA2 ex12	β-catenin IHC negative (not sequenced)	*MDM2* negative (IHC/FISH)	Intralesional excision	Local recurrence at 3 mo, 1.5 y, 7.5 y; no metastasis	Re-excision (not detailed)	Alive (10 y; s disease status not specified)
Kao et al. 2021 [8] (Case 3)	76/M	Scrotum (dermis–subcutis)	MEIS1 ex6::NCOA2 ex12	β-catenin IHC negative (not sequenced)	*MDM2* negative	Excision (primary excised years earlier)	Local recurrence (years after primary excision)	Excision	Alive (follow-up not specified)
Kao et al. 2021 [8] (Case 4)	38/F	Para-rectal; 14 cm	MEIS1 ex6::NCOA2 ex12	β-catenin IHC negative (not sequenced)	*MDM2* negative	Excision	Intrapelvic local recurrence at 4, 6, 8 y; no metastasis	Repeated excisions	Alive (12 y; disease status not specified)
Gao et al. 2025 [14] (Case 1)	24/F	Pelvic cavity; 16 cm	MEIS1 ex6::NCOA2 ex12	Wild-type (tested)	None; *MDM2* not amplified (FISH)	Surgical resection	Repeated local recurrences over 3 y (2nd at 22 mo)	6 cycles liposomal doxorubicin + ifosfamide	Alive with disease (39 mo)
Gao et al. 2025 [14] (Case 2)	58/F	Pelvic cavity (primary) + liver/gastroduodenal metastases	MEIS1 ex7::NCOA2 ex13	Mutated D32Y (in metastases only)	10q23-26 amplification (FAS, TLX1, NT5C2, SHOC2, TCF7L2, FGFR2); MDM2 not amplified	Intra-abdominal tumor resection	Liver + gastroduodenal metastases at presentation; rapid extensive abdominal recurrence	Liposomal doxorubicin (3 mo)	Dead of disease (7 mo after surgery)
Ribeiro et al. 2022 [12]	55/F	Left fallopian tube (2.5 cm primary) + omentum (17 × 17 × 8 cm)	MEIS1::NCOA2 (exons not specified)	Not reported	Final dx: Müllerian adenosarcoma with sarcomatous overgrowth; Ki-67 ~50%; WT-1 positive; CD10 variably positive; ER variably positive; desmin/myogenin/S100/CD117 negative	TAH + BSO + omentectomy + peritoneal biopsies	Diffuse peritoneal involvement + omental caking at initial presentation (not post-treatment recurrence)	Not reported	Not reported
Ramineni et al. 2025 [20]	19/F	Pelvis/omentum/mesoappendix; 14 cm	MEIS1 ex7::NCOA1 ex13	Wild-type (Mayo panel incl. CTNNB1 & BCOR)	No other alteration; no germline variant	Pelvic resection; hysterectomy (ovaries preserved).	Diffuse abdominopelvic disease at presentation; uterus/adnexa normal	—	Not reported (follow-up not reported)
Present case	38/F	Bartholin gland (right vulva); 3.5 cm	MEIS1 ex7::NCOA2 ex13	Mutated S33C (p.Ser33Cys, VAF 8.1%; β-catenin nuclear+)	*MDM2* not amplified (FISH); flat SNP-array/low genomic complexity	Excision (R1, incomplete); initial surveillance	Local recurrence at 8 mo (MRI/PET); no distant metastasis	Wide re-excision (R1) → adjuvant radiotherapy 50 Gy + MR-Linac boost	Alive; (14 mo)

Spindle-cell rhabdomyosarcomas harboring the same fusion are excluded. Cases are grouped by anatomical site and, within each group, listed chronologically by year of publication; the present case is shown last. Follow-up duration (when reported) is given in parentheses in the last column. * Only previously published MEIS1::NCOA1/2 fusion sarcoma treated with radiotherapy; given for multifocal peritoneal recurrence after resection (not as primary adjuvant therapy) and combined with doxorubicin, without preventing progression. The present case is the first to receive curative-intent adjuvant radiotherapy after R1 resection. Abbreviations: BSO, bilateral salpingo-oophorectomy; F, female; FISH, fluorescence in situ hybridization; IHC, immunohistochemistry; LVI, lymphovascular invasion; M, male; MSS, microsatellite stable; TAH, total abdominal hysterectomy; TMB, tumor mutational burden; VAC, vincristine/doxorubicin/cyclophosphamide; VAF, variant allele frequency.
